# Transcriptome Analysis Reveals Key Pathways and Hormone Activities Involved in Early Microtuber Formation of *Dioscorea opposita*

**DOI:** 10.1155/2020/8057929

**Published:** 2020-03-11

**Authors:** Junhua Li, Xiting Zhao, Yahui Dong, Shujie Li, Jiaojiao Yuan, Chenglong Li, Xiaoli Zhang, Mingjun Li

**Affiliations:** ^1^College of Life Sciences, Henan Normal University, Xinxiang, Henan 453007, China; ^2^Engineering Technology Research Center of Nursing and Utilization of Genuine Chinese Crude Drugs of Colleges and Universities in Henan Province, Xinxiang, Henan 453007, China; ^3^Engineering Laboratory of Biotechnology for Green Medicinal Plant of Henan Province, Xinxiang, Henan 453007, China

## Abstract

Chinese yam (*Dioscorea opposita*) is an important tuberous crop used for both food and medicine. Despite a long history of cultivation, the understanding of *D. opposita* genetics and molecular biology remains scant, which has limited its genetic improvement. This work presents a *de novo* transcriptome sequencing analysis of microtuber formation in *D. opposita*. We assembled cDNA libraries from different stages during the process of microtuber formation, designated as initial explants (EXP), axillary bud proliferation after three weeks (BUD), and microtuber visible after four weeks (MTV). More differentially expressed genes (DEGs) and pathways were identified between BUD vs. EXP than in MTV vs. BUD, indicating that proliferation of the axillary bud is the key stage of microtuber induction. Gene classification and pathway enrichment analysis showed that microtuber formation is tightly coordinated with primary metabolism, such as amino acid biosynthesis, ribosomal component biosynthesis, and starch and sucrose metabolism. The formation of the microtuber is regulated by a variety of plant hormones, including ABA. Combined with analysis of physiological data, we suggest that ABA positively regulates tuberization in *D. opposita*. This study will serve as an empirical foundation for future molecular studies and for the propagation of *D. opposita* germplasm in field crops.

## 1. Introduction

Yams (*Dioscorea* spp.) are a tuberous crop in many tropical and subtropical regions, such as West Africa, East and South Asia, and the Caribbean. Around ten *Dioscorea* species have been domesticated, and they are important sources of food and income in these areas. *Dioscorea opposita* (Chinese yam) is one of the four famous Chinese herbs produced in Huaiqing area, it is also a very popular edible plant and has long been cultivated to promote human health and longevity through diet, and it is the 2^nd^ most commonly grown tuberous crop in China after potato. In recent years, *D. opposita* has drawn more and more research attentions on its biology, pathology, and cultivation [1, 2].

Plant diseases, especially virus infections that resulted from vegetative propagation, are a serious issue for field production of *D. opposita*. The production of virus-free plants through plant tissue culture is widely used in the *Dioscorea* genus to avoid virus infections of plant materials [3–5]. However, the virus-free plantlets obtained by this approach are very fragile and difficult to pack, transport, and transplant. In addition to plantlets, microtubers are small tubers originated from plant tissues *in vitro*. Microtubers have the potential to be integrated into seed yam programs [6, 7] and are particularly convenient for shipping, storage, and exchange of germplasm. Therefore, the study of microtuber induction and formation has attracted more and more attention [8, 9].

Tuberization is a highly complex biological process which is affected by both environmental (such as photoperiod) and endogenous factors (such as plant growth regulators and plantlet growth stage) [1, 5, 10]. The regulation mechanism of tuberization is invaluable to devise strategies to improve tuber yield and quality. Researchers are now interested in identifying the regulatory molecules related to the formation of microtubers. The high-throughput capacity of the next generation of RNA sequencing technology provides an unprecedented opportunity for genomic exploration and gene discovery in non-model plant species for which there is no available reference genome sequence data [11, 12]. RNA-Seq results typically show high levels of reproducibility for both technical and biological replicates [13, 14]. During the past decade, the genes and gene networks associated with important potato tuber traits, such as the tuber formation [15–17], antiviral properties [18–20], and starch biosynthesis [21–23], have been identified and functionally characterized using RNA-Seq technology. So far, knowledge of plant tuberization was mainly obtained from studies in potato. The genomic resources available for *D. opposita* or for other members of the Dioscoreaceae family are limited, and the transcriptional changes and molecular mechanisms associated with the developmental process of microtubers in *D. opposita* are still far from being characterized. This lack of information hampers gene discovery and seriously hinders the improvement of *D. opposita* as a commercially important species.

A one-step protocol for induction of microtubers from a single nodal segment was previously established [9], which provides a simplified model for the study of microtuber formation in *D. opposita*. In this study, Illumina RNA-Seq technology was used to investigate changes in the transcriptome during microtuber formation in *D. opposita*. The results of this transcriptomic analysis represent a valuable bioinformatics resource for further research into the genes and gene functions involved in controlling the process of microtuber formation.

## 2. Materials and Methods

### 2.1. Plant Materials

**Figure 1:**
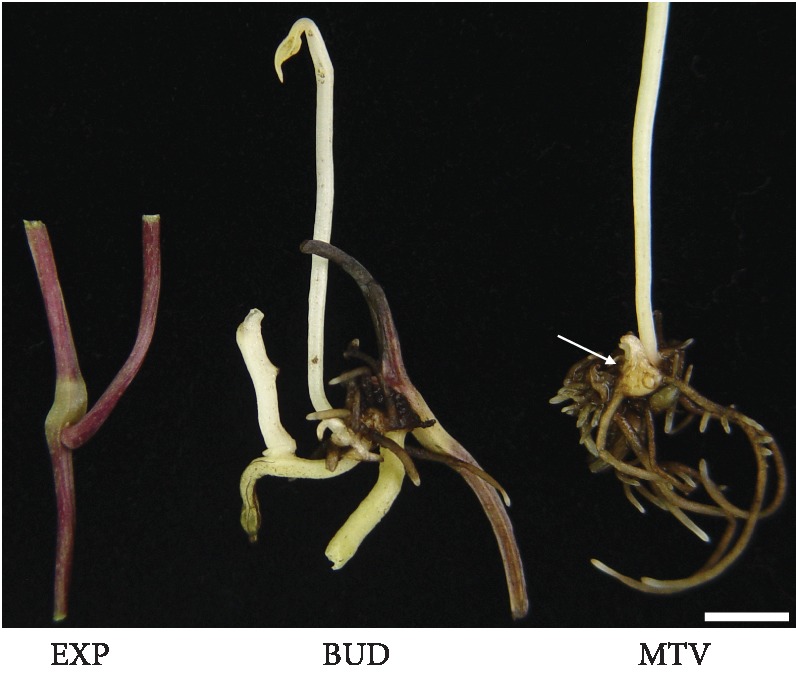
Microtuber induction and formation stages in *D. opposita*. Pictures of *D. opposita*: at the stages of initial explant (EXP), axillary bud proliferation after three weeks (BUD), and microtuber visible after four weeks (MTV). The arrow indicates a microtuber. Bar = 5 mm.

Microtubers of *D. opposita* cultivar Tiegun were collected from those plants of Henan Province Engineering Laboratory of Green Medicinal Plant Biotechnology at Henan Normal University in Xinxiang, China. Plants were then grown in a growth chamber in the liquid MS medium containing 60 g L^−1^sucrose under a 16 h light/8 h dark photoperiod with a light intensity of 38 *μ*m sec^−1^ m^−2^ at 22-25°C [9]. Three different stages of microtuber formation are shown in Figure 1. All samples were dissected from the plants, immediately frozen with liquid nitrogen, and then were put at -80°C.

### 2.2. RNA Extraction and cDNA Library Preparation for Transcriptome Sequencing

Total RNA used for RT-qPCR analysis was extracted from tissues of three stages of microtuber formation using a RNAiso for Ploysaccharide-rich Plant Tissue Kit (Takara, Dalian, China). RNA purity was checked using a NanoPhotometer® spectrophotometer (IMPLEN, USA). RNA concentrations were measured using a Qubit® RNA Assay Kit in Qubit® 2.0 Flurometer (Life Technologies, USA). RNA integrity was assessed using an RNA Nano 6000 Assay Kit with an Agilent Bioanalyzer 2100 system (Agilent Technologies, USA). A total of 3 *μ*g RNA per sample was used for library construction. For each stage, the RNA samples from three individuals were then pooled together in equal amounts to generate one mixed sample. Each stage with two replicates, then these six mixed RNA samples were subsequently used in cDNA library construction and Illumina deep sequencing.

Six cDNA libraries were generated using a NEBNext® Ultra™ RNA Library Prep Kit for the Illumina® platform (NEB, USA) following the manufacturer's recommendations; index codes were added to attribute sequences to each sample. Library quality was assessed with an Agilent Bioanalyzer 2100 system. The library preparations were sequenced with the Illumina Hiseq 2000 platform and paired-end reads were generated at Beijing Novogene Bioinformatics Biotechnology Co., Ltd. (Beijing, China). All raw-sequence reads data were deposited in NCBI Sequence Read Archive (SRA, http://www.ncbi.nlm.nih.gov/Traces/sra) with accession number SRP061414.

### 2.3. *De Novo* Transcriptome Assembly

Raw data (raw reads) in the fastq format were initially processed with in-house perl scripts. In this step, clean data (clean reads) were obtained by removing reads containing adapters, reads containing ploy-N, and low-quality reads from raw data. At the same time, the Q20 and Q30 values, the GC-content, and the sequence duplication level of the clean data were calculated. All of the downstream analyses were based on the high-quality data prepared via these initial processing procedures.

### 2.4. Sequence Annotation and Classification

For annotation, the sequences were searched against the NCBI NR protein database (https://www.ncbi.nlm.nih.gov/protein/) using the BlastX algorithm, with a cut-off *E* value of 1*e*-5. Gene Ontology (GO) terms were extracted from the annotation of high-score BLAST matches in the NCBI NR protein database using Blast2GO v2.5 [24] and then sorted for the GO categories using in-house perl scripts. Functional annotation of the proteome was carried out by a BlastP homology search against the NCBI eukaryotic Ortholog Groups (KOG) database. KEGG pathway annotations were performed using Blastall software.

### 2.5. Differential Expression Analysis

After the *D. opposita* transcriptome was assembled, counting of alignments was performed using the RSEM package [25]. Differential expression analysis of two conditions/groups was performed using the DESeq R package [26, 27]. The DESeq R package provides statistical routines for determining differential expression in digital gene expression data using a model based on a negative binomial distribution. The *P* value sets the threshold for the differential gene expression tests. The resulting *P* values were adjusted using Benjamini and Hochberg's approach for controlling the false discovery rate (FDR) [28]. Genes with an adjusted *P* value < 0.05 determined by DESeq were classified as differentially expressed. The sequences of the DEGs were Blast (BlastX) against the genome of a related species to obtain the Predicted Protein-Protein Interactions (PPI) of these DEGs. The PPI of the DEGs were visualized in Cytoscape [29]. We used KOBAS software to test the statistical enrichment of differentially expressed genes in KEGG pathways [30].

### 2.6. Real-Time Quantitative PCR to Validate the RNA-Seq Data

Nine DEGs were chosen for validation using real-time qPCR. The primers, designed with Primer Premier 5.0 software, are detailed in [Supplementary-material supplementary-material-1]. Total RNA was extracted from tissues at each stage using a TaKaRa MiniBEST Plant RNA Extraction Kit (TaKaRa, Japan) and reverse-transcribed into cDNA using HiScript® Q Select RT SuperMix for qPCR (Vazyme, USA). Real-time qPCR was performed on an ABI 7500 Real-Time PCR system (Applied Biosystems, USA). The level of expression was calculated with normalization using *Actin* as a reference [31]. A comparative Ct method (2^–*ΔΔ*Ct^) of relative quantification was used to analyze the real-time quantitative PCR data [32, 33]. All quantitative PCR analyses for each gene used three biological replicates.

### 2.7. ABA Treatment

To investigate the influence of ABA on microtuber formation, single nodal segments were cultured under the same conditions that we developed previously [9] in medium containing 1 *μ*M ABA or 0.5 mM sodium tungstate (ST). For each treatment, three independent experiments were conducted and each repetition contained 36 single nodal segments. Values represent means ± SE of three independent measurements. Student's *t*-test was used to evaluate the differences between every treatment and the control.

## 3. Results

### 3.1. Illumina Sequencing and the Assembly of Sequence Reads

In a previous study, a simplified method for *in vitro* induction of microtubers from *D. opposita* was developed, by which microtubers can be induced from almost all of the internodes of single nodal segments in MS medium under rotary shaking conditions [9]. The hormone-free induction conditions and the highly reproducible results made this method a model system for studies of the molecular mechanisms behind the process of microtuber formation, such as differences in gene expression and transcriptome profile. In order to perform an expression profiling study, here we divided the process of microtuber formation into the following three stages based on appearance changes of the leaf-axil region and the microtuber emergence (Figure 1): initial explant (EXP), axillary bud proliferation after three weeks (BUD), and microtuber visible after four weeks (MTV). Based on the present study and our previous work [9], the starting control material, microtuber induction, and microtuber formation are thought to be well-represented by materials from the above three stages.

Total RNA from these stages was extracted; cDNA libraries for each developmental stage (EXP, BUD, and MTV) were constructed and sequenced. After eliminating primer and adapter sequences and filtering out the low-quality reads, we pooled all the high-quality Illumina reads from the three different stage libraries. We obtained 50,072,432 and 53,666,222, 43,944,164 and 57,705,804, and 53,460,778 and 65,554,462 qualified Illumina reads, respectively, for the two EXP samples, the two BUD samples, and the two MTV samples, giving rise to 5.01G and 5.36G, 4.39G and 5.78G, and 5.34G and 6.56G total bases, respectively ([Supplementary-material supplementary-material-1]). Using Trinity software, we then combined all the reads to form a transcriptome database for *D. opposita* [34]. We identified 181,047 transcripts, with total residues of 234,740,027 bp. The average length of each transcript was 1,297 bp; the shortest sequence was 201 bp in length and the longest sequence was 18,739 bp in length ([Supplementary-material supplementary-material-1]).

The sequence length distribution of the unigenes and transcripts is shown in [Supplementary-material supplementary-material-1]. The unigenes have a length of less than 301 bp (21,687), representing the highest proportion (30.23%), followed by the 301-500 bp sequences (17,465, 24.35%) and 501-1000 bp sequences (15,314, 21.35%); there were 45,419 transcripts (25.09%) in the size range of 1001-2000 bp, 39,546 (21.84%) at >2000 bp, and 37,413 (20.66%) at 501-1000 bp.

### 3.2. Real-Time Quantitative PCR Validation of RNA-Seq Results

To validate the RNA-Seq results, nine DEGs were selected and RT-qPCR assays were performed. The expression patterns of these genes at each of the three stages of microtuber formation are shown in [Supplementary-material supplementary-material-1]. Although the calculated fold changes showed slightly varied, most genes measured by real-time qPCR were consistent with the RNA-Seq results. These experiments confirmed the accuracy of the RNA-Seq results reported in this study.

### 3.3. Sequence Annotation

Since there is no reference sequence data available for directly assembling the transcriptome of *D. opposita*, sequences obtained were searched against the NCBI nonredundant (NR) database using BlastX [35] with a cut-off *E* value at 10^−5^. A total of 23,310 (32.49%) sequences showed significant similarity to known proteins in the NR database. Among them, 13.89% demonstrated 80%-100% similarity as reported in the BlastX results, and 38.21% showed 60%-80% of RNA sequence similarity ([Supplementary-material supplementary-material-1]). After a closer look of the specific origins of the matched sequences, we found that 13.53% of the sequences matched to the transcripts of *Vitis vinifera*, which was followed by *Oryza sativa* (13.05%) and *Arabidopsis* (11.19%) ([Supplementary-material supplementary-material-1]).

### 3.4. GO, KOG, and KEGG Classification

**Figure 2:**
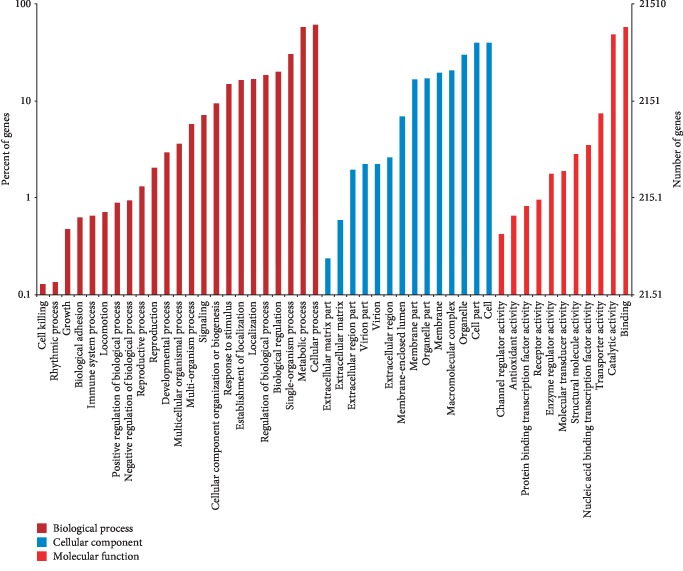
Gene Ontology (GO) assignments for the microtuber induction and formation transcriptome of *D. opposita*. Results are summarized under three main GO categories: biological process, cellular component, and molecular function. The left *y*-axis indicates the percentage of a specific subcategory of genes in each main category. The right *y*-axis indicates the number (count) of genes.

To address the gene product properties, we adopted Gene Ontology (GO) terms using Blast2GO v2.5 [24], which annotates high-score BLAST matches to sequences in the NCBI NR protein database. Genes of *D. opposita* were classified into biological process, cellular component, and molecular function GO categories and 48 subcategories. Of the 181,047 assembled transcripts, 21,514 were successfully annotated with GO assignments; some of these belonged to one or more of the three categories (Figure 2). The “biological process” categories included cellular process (13,117, 60.97%), metabolic process (12,416, 57.71%), single-organism process (6,549, 30.44%), biological regulation (4,328, 20.12%), regulation of biological process (3,977, 18.49%), and other categories (18,347, 85.28%). The major proportion of the “cellular component” categories included cell (8,582, 39.89%), cell part (8,556, 39.77%), organelle (6,478, 30.11%), macromolecular complex (4,464, 20.75%), membrane (4,209, 19.56%), organelle part (3,672, 17.07%), and membrane part (3,579, 16.64%). The top three most abundant subcategories of the “molecular function” category were binding (12,455, 57.89%), catalytic activity (10,407, 48.37%), and transporter activity (1,602, 7.45%).

**Figure 3:**
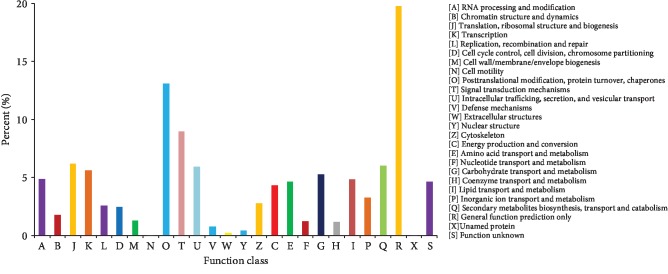
KOG functional classification for the microtuber induction and formation transcriptome of *D. opposita*. From a total of 181,047 *de novo* assembled transcripts, 9,404 transcripts with significant homologies in the KOG database (*E* value ≤ 1*e* − 3) were classified into 26 KOG categories.

Identification of orthologous groups is useful for genome annotation. The annotated sequences were subjected to a search against the eukaryotic Ortholog Groups (KOG) database [36], for functional prediction and classification. Based on sequence homology, 9,404 unique sequences were assigned a KOG functional classification. These sequences were classified into 26 KOG categories involved in cellular process, signal transduction, metabolism, and other processes (Figure 3). The most common category was the nonspecific category of “general function prediction only” (1,859, 19.77%), followed by “posttranslational modification, protein turnover, and chaperones” (1,230, 13.08%), “signal transduction mechanisms” (843, 8.96%), “translation, ribosomal structure, and biogenesis” (580, 6.17%), “secondary metabolite biosynthesis, transport, and catabolism” (566, 6.02%), and “intracellular trafficking, secretion, and vesicular transport” (555, 5.90%). Among other categories represented were “translation” (529, 5.63%) and “carbohydrate transport and metabolism” (496, 5.27%). The least well-represented categories were “cell motility” (3, 0.03%) and “unnamed protein” (1, 0.01%).

**Figure 4:**
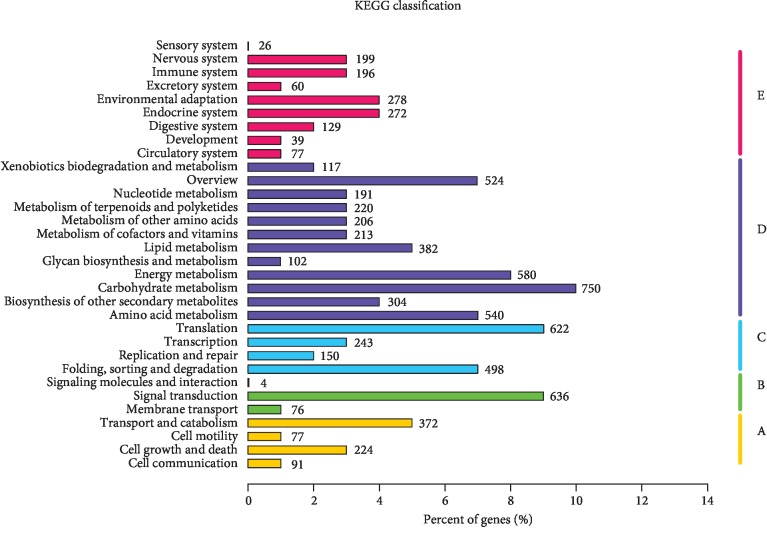
KEGG functional classification for the microtuber induction and formation transcriptome of *D. opposita*. From a total of 181,047 *de novo* assembled transcripts, 7,278 transcripts with significant homologies in the KEGG database were classified into 5 KEGG categories. A: cellular processes; B: environmental information processing; C: genetic information processing; D: metabolism; and E: organismal systems.

In order to identify biochemical pathways, we mapped the annotated sequences onto the KEGG (Kyoto Encyclopedia of Genes and Genomes) database [30], which is an alternative approach to predict and assign gene function by emphasizing biochemical pathways (Figure 4). A total of 7,278 annotated genes were assigned to 262 KEGG pathways. The maps with the highest unigene representation were carbohydrate metabolism (750, 10.31%), followed by signal transduction (636, 8.74%) and translation (622, 8.55%). The pathways with the highest representation were carbon metabolism (315, 4.33%), biosynthesis of amino acids (277, 3.81%), ribosome (251, 3.45%), and plant hormone signal transduction (223, 3.06%). These pathway assignments can provide valuable information for the investigations focused on specific biochemical and developmental processes.

### 3.5. Differentially Expressed Genes (DEGs) during Microtuber Formation

**Figure 5:**
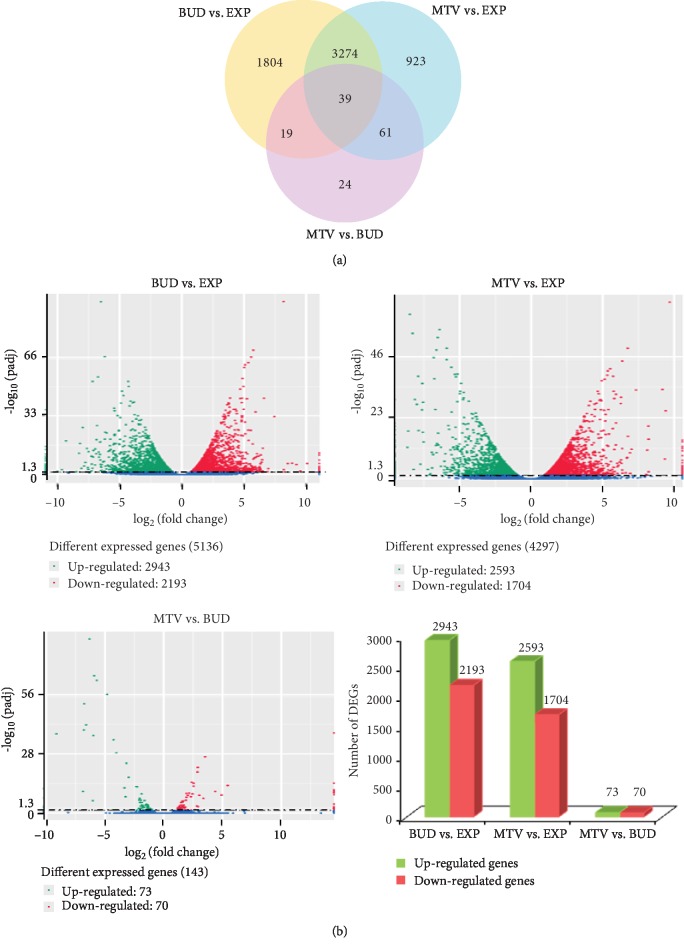
Differentially expressed genes (DEGs) in the microtuber formation-stage comparisons. (a) DEG Venn diagram. The sum of the numbers in each large circle represents the total number of DEGs of the comparative combination, and the circle overlapping part represents what the DEG combination has in common. (b) Whole-study overview of log-fold changes in gene expression in comparisons BUD vs. EXP (upper left), MTV vs. EXP (upper right), and MTV vs. BUD (down left). The *x*-axis indicates the log-fold changes between the two samples. The *y*-axis indicates the absolute expression levels (–log_10_ (Padj)). The number of up- or downregulated genes in BUD vs. EXP, MTV vs. EXP, and MTV vs. BUD are shown in the down-right panel.

We mapped the Illumina reads from each stage (EXP, BUD, and MTV) onto our assembled transcriptome database. The expression of each gene was calculated using the number of reads that mapped onto a given transcript. The DEG Venn diagram of the comparisons is depicted in Figure 5(a). It shows 1,804 and 923 genes are specific differentially expressed in BUD vs. EXP and MTV vs. EXP comparisons during microtuber formation, respectively.

There were 5,136, 4,297, and 143 genes differentially expressed (*P* < 0.05) between sample pairs of BUD-EXP, MTV-EXP, and MTV-BUD, respectively. The expression patterns of the differentially expressed genes (DEGs) from the EXP vs. BUD are depicted in Figure 5(b); there were 2,943 genes that are shown to be 10-fold greater and 2,193 genes that are shown to be 9-fold weaker in BUD vs. EXP. The expression patterns of DEGs from the MTV vs. EXP comparison are depicted in Figure 5(b); there were 2,593 genes that are shown to be 9-fold greater and 1704 genes that are shown to be 8-fold weaker in MTV vs. EXP.

To further investigate genes that were highly correlated with microtuber formation, we looked into the 20 most upregulated and the 20 most downregulated genes selected from the BUD vs. EXP, MTV vs. EXP, and MTV vs. BUD comparisons. Some of these genes showed matches to sequences in the NCBI NR database, such as fructose-bisphosphatealdolase, chlorophyll a/b-binding protein type III precursor, protein LHY-like, glycosyltransferase GT14A05, and Chain A, Banana Lectin ([Supplementary-material supplementary-material-1]).

### 3.6. Pathway Enrichment Analysis of DEGs

In order to explore the functions of the DEGs in greater depth, the KEGG Orthology-Based Annotation System (KOBAS) [37, 38] was used. Among these pathways, the terms that were found to be significantly differently expressed (corrected *P* value ≦ 0.05) in both the BUD vs. EXP and the MTV vs. EXP comparisons were involved in “glycolysis/gluconeogenesis” and the “citrate cycle” (TCA cycle). We noticed a high percentage of significantly DEGs of the biosynthesis of secondary metabolites, microbial metabolism in diverse environments, carbon metabolism, and ribosome pathways (see Discussion). We found that the only pathway enriched in MTV vs. BUD comparison was the photosynthesis-antenna protein pathway, which means DEGs of BUD vs. EXP and MTV vs. EXP comparisons may play a more important role during microtuber formation (Table 1). Then, the KEGG enrichment on the specific DEGs of BUD vs. EXP and MTV vs. EXP comparisons was conducted. We found that the genes involved in biosynthesis of amino acids, followed by carbon metabolism and phenylpropanoid biosynthesis pathways, were significantly enriched in BUD, suggesting these pathways represent key metabolic processes during microtuber induction. Our data also suggested that ribosome, photosynthesis and the metabolism of amino sugar, and nucleotide sugar play roles in microtuber formation ([Supplementary-material supplementary-material-1]).

Tubers are starch-rich storage organs, in which large amounts of starch are deposited. In potato, studies showed that expression of the sucrose synthase gene and genes encoding enzymes involved in starch biosynthesis were increased during tuber formation [39–41]. Microtubers of *D. opposita* likely share the same fundamental mechanism of its development as do potato and other tuberous plants. Given this hypothesis, we examined gene activities of the starch and sucrose metabolism pathways. Our data showed that almost all of the key genes in these pathways were found among the DEGs, including nineteen starch and sucrose metabolic genes in the BUD vs. EXP comparison and sixteen genes in the MTV vs. BUD comparison ([Supplementary-material supplementary-material-1]). We also found that there were no DEGs in the starch or sucrose metabolism pathways in the MTV vs. BUD comparison.

### 3.7. Differentially Expressed Genes Involved in Plant Hormone Signaling Pathways during Microtuber Formation

A group of genes involved in plant hormone activities were upregulated at BUD and MTV compared to EXP ([Supplementary-material supplementary-material-1]). Those genes include *ARF*, *GH3*, *AHP*, *ARR*, *PP2C*, *JAZ*, and *TGA*. Among the upregulated genes, the ethylene-responsive genes *EIN3* and *CTR1* were significantly upregulated at BUD while the ABA-responsive gene *PP2C* was significantly upregulated at MTV. These results indicate that the formation of the microtuber is coordinated and regulated by a variety of hormones, such as ethylene and ABA.

### 3.8. ABA Is a Positive Regulator of Microtuber Formation

**Figure 6:**
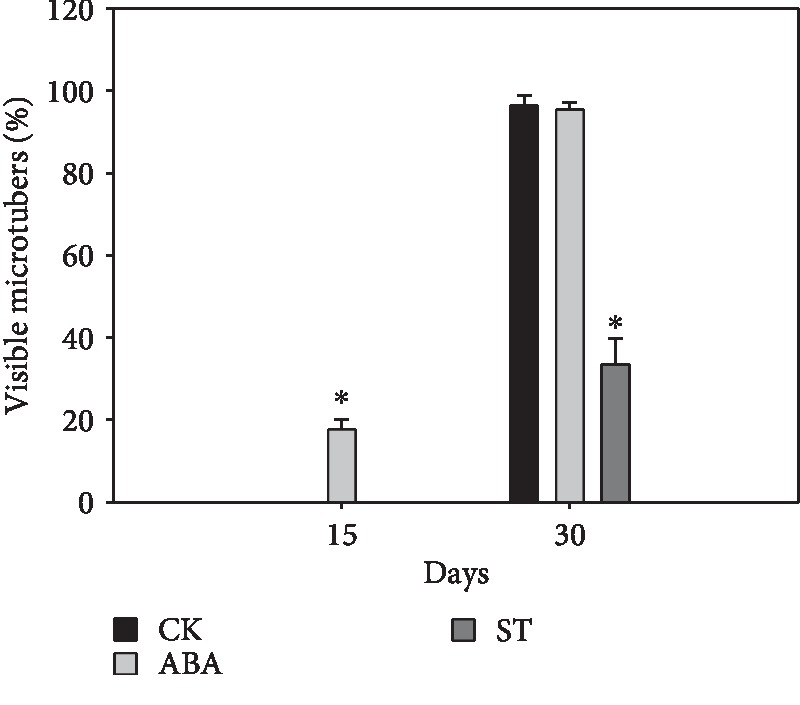
The influence of exogenous ABA on the rates of microtuber formation. ST: sodium tungstate. Data are presented as means ± SD. Asterisks indicate significance (^∗^*P* < 0.01 versus control, Student's *t*-test).

Transcriptional profiling showed that microtuber formation in *D. opposita* is associated with altered transcription state of *PP2C*, which has both positive and negative roles in ABA signaling [42]. We therefore carried out further studies to elucidate the role of ABA in microtuber formation. We tested the influence of ABA and ST (an ABA inhibitor) on microtuber formation. The formation of microtubers could be observed on 17.6% of single nodal segments on day 15 in medium with 1 *μ*M ABA, which is obviously earlier than that of the control (Figure 6). In contrast, the percentage of microtuber formation was reduced three times compared with the control on day 30, when microtubers were formed in most single nodal segments of the control and the ABA-treat group (Figure 6). In addition, the endogenous levels of ABA were measured in different stages of microtuber formation in our previous study [43], in which the ABA contents showed a positive association with the process of microtuber formation, which reached its maximum level at the MTV stage, and maintained at a high level thereafter [43]. These results indicate that ABA had a positive role in regulating microtuber formation.

## 4. Discussion

### 4.1. Transcriptomic Study Provides a Basis for Further Research of Microtuber Formation in *D. opposita*

*D. opposita* is an important tuberous crop for both food and medicine. However, a limited genetic resource, including genomic information, is available for this species, which has limited its genetic improvement. In this study, we generated cDNA sequence data, comprising long sequences of good quality, which will facilitate subsequent and more detailed studies. The analysis included sample cDNA libraries from three different stages of microtuber formation. We identified a total of 71,739 unigenes, and the sequences were annotated against the NCBI NR database using BlastX. By using Illumina's digital gene expression platform, we investigated differential gene expression during the formation of *D. opposita* microtubers and analyzed the expression of unigenes in the context of the pathways of the KEGG. We found biosynthesis of amino acids and ribosome may represent key metabolic processes during microtuber induction and formation, respectively. We identified biosynthesis of amino acids and ribosome pathway genes that were differentially expressed during microtuber induction and formation. We also found that key genes in the metabolism of starch and sucrose were differentially expressed during the various stages of microtuber formation. To our knowledge, this is the first comprehensive transcriptomic study of *D. opposita* to identify genes and pathways that are differentially expressed during microtuber formation; the results are foundational for functional genomic studies in this species.

For gene annotation, the sequences were searched against the NCBI NR database. Because of the limited genetic information available, no matches were obtained for as many as 48,429 (67.51%) of the sequences. Some of these sequences may represent novel genes in *D. opposita*, suggesting that some unique processes and pathways might be involved with microtuber formation in *D. opposita*. Interestingly, the annotated sequences of *D. opposita* shared relatively high homology with *Vitis vinifera* proteins ([Supplementary-material supplementary-material-1]). In spite of the large proportion of sequences that showed no matches, a large number of transcripts were nevertheless assigned to a wide range of GO and KOG classifications, which indicated that our transcriptome data represented a broad diversity of transcripts in *D. opposita*. KEGG functional classification identified many genes associated with carbon metabolism, including genes of glycolysis/gluconeogenesis, the citrate cycle (TCA cycle), fructose and mannose metabolism, galactose metabolism, pentose biosynthesis, and inositol phosphate metabolism. Additionally, we identified genes related to starch and sucrose metabolism. Starch composition is one of the most economically important characteristics of *D. opposita*, since tubers and fully matured microtubers are a rich source of energy, and starch constitutes most of the dry weight of the microtubers.

### 4.2. DEGs Revealed Pathways Enriched during Microtuber Formation

Although the analysis of DEGs during microtuber formation has been performed using microarray technology in potato [16], the availability of data relating to the transcriptomes of microtuber formation at different stages in *D. opposita* remains vacant. Here, the analysis of DEGs during microtuber formation has been performed using Illumina RNA-Seq method microarray in *D. opposita*, and a number of DEGs were identified, which showed differential expression patterns in the following comparisons: BUD vs. EXP, MTV vs. EXP, and BUD vs. MTV.

Analysis of DEGs showed that there was only one pathway enrichment in BUD vs. MTV comparison. Therefore, DEGs of BUD vs. EXP and MTV vs. EXP comparisons may play an important role during microtuber formation ([Supplementary-material supplementary-material-1]). Through analyzing the KEGG enrichment data on the DEGs of BUD vs. EXP and MTV vs. EXP comparisons, we found that the biosynthesis of amino acids, carbon metabolism, and phenylpropanoid biosynthesis is significantly differently expressed (corrected *P* value ≦ 0.05) in the BUD vs. EXP and speculated that the biosynthesis of amino acids, followed by carbon metabolism and phenylpropanoid biosynthesis, may play key regulatory roles during microtuber induction of *D. opposita*. Our results are consistent with those of Roessner-Tunali et al. [44], who linked sucrose levels to the regulation of *de novo* amino acid synthesis at the transcriptional level. A possible explanation for these results may be an elevated requirement for free amino acid levels to maintain cellular osmoticum during tuber induction [45].

The ribosome pathway appears to play an important role during microtuber formation of *D. opposita*. In addition, photosynthesis and metabolism of amino sugars and nucleotide sugars also appear to have a supporting function in these processes.

### 4.3. Potential Roles of Starch Formation and Hormone Activities in Microtuber Formation

As storage organs, large amounts of starch are deposited in tubers. Our data showed that key genes in starch and sucrose metabolic pathways are associated with early microtuber induction. In our previous study, microtubers could be easily induced in a high sugar medium [9]. It is conceivable that the availability of starch or one of its precursors acts as a signal, to initiate the morphological typical changes not only for tuberization but also for the activation of the “tuber-specific” genes and early microtuber induction.

Transcriptome data indicated that microtuber formation is regulated by a variety of hormones, such as ethylene and ABA, since multiple genes involved in ethylene and ABA activities were upregulated at BUD and MTV ([Supplementary-material supplementary-material-1]). The results are consistent with our previous study, in which the ABA content increased during microtuber formation [43]. We showed that exogenous ABA and its inhibitor significantly influenced microtuber formation and concluded that ABA plays a positive role in this process. It is well known that ABA functions directly in plant responses to stress (e.g., [46]); therefore, in *D. opposita*, ABA might be an internal signal integrator through which plants can sensitively perceive incoming stresses and regulate their physiology accordingly (such as the production of microtubers). In summary, our results not only advance our understanding of early tuberization but also are potentially valuable for future mechanism studies, e.g., roles of other plant hormones in microtuber formation of *D. opposita*.

## 5. Conclusions

The transcriptome sequencing analysis of microtuber formation indicates that the proliferation of axillary bud is the key stage of microtuber induction. DEGs involved in starch and sucrose metabolism during microtuber formation were identified. We suggest that ABA positively regulates tuberization in *D. opposita*.

## Figures and Tables

**Table 1:** Significantly enriched pathways identified using the KOBAS database based on DEGs identified during microtuber formation.

			BUD vs. EXP			MTV vs. EXP			MTV vs. BUD		
Pathway	Pathway ID	Bn	Nt	*P* value	Corrected *P* value	Nt	*P* value	Corrected *P* value	Nt	*P* value	Corrected *P* value
Photosynthesis-antenna proteins	ko00196	39							7	3.36*E*-10	5.94*E*-08
Flavonoid biosynthesis	ko00941	81	35	5.07*E*-07	8.97*E*-05						
Ribosome	ko03010	251				104	0	0			
Glycolysis/gluconeogenesis	ko00010	145	51	3.27*E*-06	0.000289	45	6.34*E*-06	0.000561			
Phenylalanine metabolism	ko00360	110	41	5.76*E*-06	0.00034						
Citrate cycle (TCA cycle)	ko00020	73	27	0.000252	0.008928	24	0.000341	0.020113			
Phenylpropanoid biosynthesis	ko00940	163	50	0.000236	0.008928						
Microbial metabolism in diverse environments	ko01120	418	107	0.000518	0.01477						
Flavone and flavonol biosynthesis	ko00944	27	13	0.000584	0.01477						
Carbon metabolism	ko01200	296	79	0.000741	0.016394						
Biosynthesis of secondary metabolites	ko01110	1104	250	0.001075	0.019019						
Butanoate metabolism	ko00650	22	11	0.001043	0.019019						
Steroid biosynthesis	ko00100	27				12	0.000511	0.022627			
DNA replication	ko03030	37				14	0.001257	0.044508			

Note: Bn (background number) indicates the total number of transcripts present in each pathway. Nt (number of transcripts) indicates the number of DEGs in each pathway. Pathways enrichment analysis with corrected *P* value ≤ 0.05 were included.

## Data Availability

The datasets supporting the results of this article are included in the article and the supplemental files accessible through the journal website. The raw sequencing datasets supporting the results of this article were deposited in the NCBI SRA repository [http://www.ncbi.nlm.nih.gov/sra?term = SRP061414].

## Abbreviations

BUD: Axillary bud proliferation
EXP: Initial explants
GO: Gene Ontology
KOG: EuKaryotic Ortholog Groups
KEGG: Kyoto Encyclopedia of Genes and Genomes
MTV: Microtuber visible after four weeks
NR: Nonredundant
DEGs: Differentially expressed genes
KOBAS: KEGG Orthology-Based Annotation System.

## Supplementary Materials

Supplementary Materials Figure S1: sequence-length distributions of unigenes and transcripts assembled from the Illumina reads. Figure S2: verification of RNA-Seq results by qRT-PCR. Figure S3: result summary for sequence-homology searching against the NCBI NR database. Table S1: summary of Illumina transcriptome sequencing for microtuber formation of D. opposita. Table S2: description of the 20 most up- and downregulated genes in the BUD vs. EXP comparison. Table S3: description of the 20 most up- and downregulated genes in the MTV vs. EXP comparison. Table S4: description of the 20 most up- and downregulated genes in the MTV vs. BUD comparison. Table S5: KEGG enriched pathways of specificity DEGs identified during microtuber induction and formation. Table S6: differentially expressed genes related to starch and sucrose metabolism during microtuber formation. Table S7: differentially expressed genes related to plant hormone signal transduction pathways during microtuber formation. Table S8: the primers used for the analysis of gene expression by qRT-PCR.
