# Supplementary material for: Transcriptome Analysis Reveals Key Pathways and Hormone Activities Involved in Early Microtuber Formation of Dioscorea opposita
Source: Biomed Res Int. 2020 Mar 11;2020:8057929. doi: 10.1155/2020/8057929 (PMC7086419; doi:10.1155/2020/8057929)
Supplement: Supplementary Materials — Figure S1: sequence-length distributions of unigenes and transcripts assembled from the Illumina reads. Figure S2: verification of RNA-Seq results by qRT-PCR. Figure S3: result summary for sequence-homology searching against the NCBI NR database. Table S1: summary of Illumina transcriptome sequencing for microtuber formation of D. opposita. Table S2: description of the 20 most up- and downregulated genes in the BUD vs. EXP comparison. Table S3: description of the 20 most up- and downregulated genes in the MTV vs. EXP comparison. Table S4: description of the 20 most up- and downregulated genes in the MTV vs. BUD comparison. Table S5: KEGG enriched pathways of specificity DEGs identified during microtuber induction and formation. Table S6: differentially expressed genes related to starch and sucrose metabolism during microtuber formation. Table S7: differentially expressed genes related to plant hormone signal transduction pathways during microtuber formation. Table S8: the primers used for the analysis of gene expression by qRT-PCR. [file 8057929.f1.zip › Supplementary figures.doc]

Supplementary figures of

**Transcriptome analysis reveals key pathways and hormone activities involved** **in early** **microtuber formation of *Dioscorea opposita***

**Figure S1 Sequence-length distributions of Unigenes and transcripts assembled from the Illumina reads.** All of the Illumina reads for each microtuber formation stage (see Figure. 1) were combined together, yielding a total of 71739 unigenes and 181047 transcripts. The horizontal and vertical axes show the length interval and the numbers of unigenes and transcripts, respectively.

**Figure S2 Verification of RNA-seq results by qRT-PCR.**

(A) Expression patterns detected by RNA-seq. (B) Expression patterns confirmed by q-PCR. The level of expression was calculated with normalization using *Actin* gene as a reference. All quantitative PCR analyses for each gene used three biological replicates, with three technical replicates per experiment; the error bars indicate SD.

**Figure S3 Results summary for sequence-homology searching against the NCBI NR database.** (A) Similarity distribution of the closest BLASTX matches for each sequence. (B) A species-based distribution of BLASTX matches for sequences. We used all plant proteins in the NCBI NR database in performing the homology search.
