# Supplementary material for: Transcriptome Analysis Reveals Key Pathways and Hormone Activities Involved in Early Microtuber Formation of Dioscorea opposita
Source: Biomed Res Int. 2020 Mar 11;2020:8057929. doi: 10.1155/2020/8057929 (PMC7086419; doi:10.1155/2020/8057929)
Supplement: Supplementary Materials — Figure S1: sequence-length distributions of unigenes and transcripts assembled from the Illumina reads. Figure S2: verification of RNA-Seq results by qRT-PCR. Figure S3: result summary for sequence-homology searching against the NCBI NR database. Table S1: summary of Illumina transcriptome sequencing for microtuber formation of D. opposita. Table S2: description of the 20 most up- and downregulated genes in the BUD vs. EXP comparison. Table S3: description of the 20 most up- and downregulated genes in the MTV vs. EXP comparison. Table S4: description of the 20 most up- and downregulated genes in the MTV vs. BUD comparison. Table S5: KEGG enriched pathways of specificity DEGs identified during microtuber induction and formation. Table S6: differentially expressed genes related to starch and sucrose metabolism during microtuber formation. Table S7: differentially expressed genes related to plant hormone signal transduction pathways during microtuber formation. Table S8: the primers used for the analysis of gene expression by qRT-PCR. [file 8057929.f1.zip › Table S1, S5-S8.doc]

**Table S1** **Summary of Illumina transcriptome sequencing for microtuber formation of *D. opposita.***

|  | EXP | | BUD | | MTV | |
| --- | --- | --- | --- | --- | --- | --- |
|  | EXP_1 | EXP_2 | BUD_1 | BUD_2 | MTV_1 | MTV_2 |
| Base number (G) | 5 | 5.36 | 4.4 | 5.78 | 5.34 | 6.56 |
| Total residues (bP) |  |  | 234740027 | |  |  |
| Number of transcripts (n) |  |  | 181047 | |  |  |
| Average length of transcripts (bp) |  |  | 1297 | |  |  |
| Average length of Unigenes (bp) |  |  | 826 | |  |  |
| Largest transcripts (bp) |  |  | 18739 | |  |  |
| Smallest transcripts (bp) |  |  | 201 | |  |  |
| Number of Unigenes (n) |  |  | 71739 | |  |  |

**Table S5 KEGG enriched pathways of specificity DEGs identified during microtuber induction and formation.**

| BUD vs EXP | | MTV vs EXP | |
| --- | --- | --- | --- |
| Pathway definition | Number of sequences | Pathway definition | Number of sequences |
| Biosynthesis of amino acids | 38 | Ribosome | 45 |
| Carbon metabolism | 26 | Photosynthesis | 15 |
| Phenylpropanoid biosynthesis | 22 | Metabolism ofamino sugar and nucleotide sugar | 13 |

**Table S6** Differentially expressed genes related to starch and sucrose metabolism during microtuber formation.

|  |  |  | BUD vs EXP | | MTV vs EXP | |
| --- | --- | --- | --- | --- | --- | --- |
| Gene ID | Putative function | Nearest match | log_2_FC | padj | log_2_FC | padj |
| comp69667_c1 | sucrose metabolic process // starch metabolic process | XP_002300758.1 | –3.8955 | 3.44E–22 | –4.5949 | 3.14E–21 |
| comp83411_c1 | starch metabolic process // sucrose metabolic process | AFS65091.1 | –2.8121 | 5.72E–21 |  |  |
| comp78830_c1 | starch metabolic process // sucrose metabolic process | NP_001042865.1 | 4.4498 | 8.77E–20 |  |  |
| comp84055_c0 | sucrose metabolic process // starch metabolic process | XP_002266321.2 | 2.2248 | 8.76E–12 | 2.1283 | 0.000511 |
| comp69667_c0 | starch metabolic process // sucrose metabolic process | XP_002307668.1 | –4.1256 | 1.31E–09 | –6.5778 | 6.12E–14 |
| comp84839_c0 | sucrose metabolic process // starch metabolic process | XP_002274716.1 | –3.6047 | 2.27E–08 | –4.4961 | 4.64E–11 |
| comp86916_c0 | sucrose metabolic process // starch metabolic process | ACJ02103.1 | 3.8063 | 1.25E–21 | 4.036 | 2.53E–28 |
| comp67962_c0 | starch metabolic process // sucrose metabolic process | BAJ85551.1 |  |  | –3.4104 | 0.008687 |
| comp27406_c0 | sucrose metabolic process // starch metabolic process | EEE69076.1 |  |  | 3.7805 | 0.010498 |
| comp80124_c0 | sucrose metabolic process // starch metabolic process | NP_001049358.1 | 3.3066 | 7.96E–12 | 3.6748 | 3.54E–22 |
| comp69423_c0 | sucrose metabolic process // starch metabolic process | XP_003576649.1 | –3.5515 | 1.56E–06 | –4.329 | 1.49E–07 |
| comp86484_c0 | sucrose metabolic process // starch metabolic process | XP_002330531.1 | 2.8915 | 0.000327 | 2.6521 | 9.76E–08 |
| comp76893_c0 | sucrose metabolic process // starch metabolic process | XP_002514210.1 | 2.7818 | 4.63E–16 | 2.8159 | 1.40E–07 |
| comp54935_c0 | sucrose metabolic process // starch metabolic process | XP_002444884.1 | 2.7987 | 0.000915 | 3.088 | 4.66E–05 |
| comp80120_c0 | starch metabolic process // sucrose metabolic process | XP_002300758.1 | 3.0862 | 0.001548 | 2.7427 | 2.12E–14 |
| comp87144_c0 | starch metabolic process // sucrose metabolic process | ACN67534.1 | –2.0011 | 0.001759 |  |  |
| comp72009_c0 | sucrose metabolic process // starch metabolic process | O04887.1 | 3.7036 | 0.002141 | 3.7278 | 1.02E–06 |
| comp39078_c0 | starch metabolic process // sucrose metabolic process | XP_002284784.1 | 3.6325 | 0.002392 |  |  |
| comp71163_c0 | starch metabolic process// sucrose metabolic process | DAA63553.1 | 3.1426 | 0.003209 | 2.8257 | 0.032311 |
| comp78783_c0 | sucrose metabolic process // starch metabolic process | XP_002321214.1 | 2.1692 | 0.00384 |  |  |
| comp67972_c0 | starch metabolic process // sucrose metabolic process | AFB77929.1 |  |  | 2.7144 | 6.37E–11 |
| comp67410_c0 | starch metabolic process // sucrose metabolic process | XP_002468329.1 | –3.4971 | 0.019074 |  |  |

Note: Differentially expressed genes with Log_2_foldchanges≥2 or ≤–2, and padj≤0.05 were included.

**Table S7 Differentially expressed genes related to plant hormone signal transduction pathways during microtuber formation.**

|  |  |  | BUD vs EXP | | MTV vs EXP | | MTV vs BUD | |
| --- | --- | --- | --- | --- | --- | --- | --- | --- |
| Gene ID | Putative function | Nearest match | log_2_FC | padj | log_2_FC | padj | log_2_FC | padj |
|  | **Auxin signal transduction** |  |  |  |  |  |  |  |
| comp73032_c0 | Auxin | BAH01249.1 | 2.8724 | 2.67E-21 | 3.0954 | 4.6121E-16 |  |  |
| comp72555_c0 | Auxin | NP_001137108.1 | 4.1882 | 2.84E-11 | 2.5963 | 0.016216 |  |  |
| comp69927_c0 | Auxin | CBI32752.3 | -4.8474 | 1.17E-09 | -2.8557 | 6.87E-05 |  |  |
| comp76554_c0 | Aux / IAA | ADG57980.1 | -7.4993 | 1.42E-13 | -4.121 | 4.00E-09 |  |  |
| comp84264_c0 | auxin response factor (ARF ) | CBA11993.1 | -2.741 | 0.000271 |  |  |  |  |
| comp75119_c1 | auxin influx carrier protein (AUX1, LAX) | CCF23026.1 | -3.4402 | 0.000143 | -3.2126 | 0.00033 |  |  |
| comp73985_c0 | auxin influx carrier protein (AUX1, LAX) | XP_003516350.1 | -3.3151 | 0.002999 | -2.7165 | 0.012837 |  |  |
| comp73985_c1 | auxin influx carrier protein (AUX1, LAX) | BAD82312.1 | -3.6221 | 0.023419 |  |  |  |  |
| comp67491_c1 | Auxin-responsive GH3 family protein (GH3) | NP_179101.1 | 6.505 | 1.74E-15 | 5.3807 | 5.01E-13 |  |  |
| comp86039_c0 | Auxin-responsive GH3 family protein (GH3) | XP_002517964.1 | -2.3068 | 3.62E-15 | -2.0967 | 2.39E-08 |  |  |
| comp67491_c0 | Auxin-responsive GH3 family protein (GH3) | ACF83491.1 | 6.0539 | 5.69E-11 | 4.9182 | 0.001494 |  |  |
| comp66044_c0 | Auxin-responsive GH3 family protein (GH3) | XP_002283886.1 |  |  | 4.4867 | 1.75E-09 | 4.9249 | 3.05E-09 |
|  | **CTK signal transduction** |  |  |  |  |  |  |  |
| comp51169_c0 | Histidine phosphotransfer protein (AHP4) | XP_002456341.1 | -5.0218 | 0.000926 | -3.2288 | 0.013691 |  |  |
| comp82669_c0 | Two-component response regulator(ARR-B) | XP_003570765.1 | 2.4481 | 8.13E-12 | 2.3333 | 1.48E-07 |  |  |
| comp79669_c0 | Two-component response regulator (ARR1) | XP_002283159.1 |  |  | 3.402 | 9.42E-18 | 2.5482 | 8.56E-15 |
| comp72765_c0 | Two-component response regulator (ARR2) | XP_002283159.1 |  |  | 2.1601 | 1.52E-06 |  |  |
|  | **ABA signal transduction** |  |  |  |  |  |  |  |
| comp83176_c0 | type 2C protein phosphatases (PP2C) | ABF70120.1 |  |  | 2.4278 | 0.009805 |  |  |
|  | **Ethylene signal transduction** |  |  |  |  |  |  |  |
| comp68230_c0 | EIN3 | XP_003601983.1 | -2.6382 | 0.000726 |  |  |  |  |
| comp64007_c0 | CTR1 | XP_002313412.1 | -2.5673 | 3.40E-17 |  |  |  |  |
|  | **Brassinosteroid signal transduction** |  |  |  |  |  |  |  |
| comp86484_c0 | Serine/threonine-protein  kinase (BRI1) | XP_002330531.1 | 2.8915 | 0.000327 | 2.6521 | 9.76E-08 |  |  |
| comp51243_c4 | Serine/threonine-protein  kinase (BRI1) | XP_003635332.1 | -5.3258 | 0.001206 | -4.275 | 0.003637 |  |  |
|  | **JA signal transduction** |  |  |  |  |  |  |  |
| comp79717_c0 | jasmonates ZIM-domain  protein (JAZ) | CBI26848.3 | 2.0633 | 8.23E-10 |  |  |  |  |
|  | **SA signal transduction** |  |  |  |  |  |  |  |
| comp79164_c0 | transcription factor TGA | CBI30045.3 | 5.1616 | 1.23E-05 | 5.2911 | 0.010158 |  |  |
| comp73376_c0 | pathogenesis-related protein 1 (PR1) | AAB09084.1 | 2.6919 | 1.28E-20 |  |  |  |  |
| comp67940_c0 | Pathogenesis-related protein 1 (PR1) | AEV89264.1 | 2.144 | 1.31E-14 |  |  |  |  |
| comp53767_c0 | Pathogenesis-related protein 1 (PR1) | AAB09084.1 | 2.3568 | 1.25E-07 |  |  |  |  |
| comp71854_c0 | Pathogenesis-related protein 1 (PR1) | ACF06599.1 |  |  | 5.935 | 9.68E-22 |  |  |

Note: Differentially expressed genes with Log2fold changes≥2 or ≤-2, and padj≦0.05 were included.

**Table S8 The primers used for the analysis of gene expression by qRT-PCR.**

| **Gene name** | **Primer** | **sequence(5'to 3')** |
| --- | --- | --- |
| comp67972_c0 | Forward | ATGTGATAAGGATAAGGGAAGG |
|  | Reverse | ACCGCTAAGGTGGCAGAC |
| comp86916_c0 | Forward | GATCAACAGGTGCAACATCGAG |
|  | Reverse | CGGCGTCACCAAAGATGAAG |
| comp78830_c1 | Forward | AAGTTAGTGCTGGTTTGTATGAGG |
|  | Reverse | TCCCGCCGTGTTTCTTATC |
| comp80120_c0 | Forward | CGTTTGGAAAGTGGTGTAGAACTC |
|  | Reverse | CTGATTCGAGTATTTTCCCCGAC |
| comp80124_c0 | Forward | GAATTCGGCGACTGAACCTTAC |
|  | Reverse | GTTGCAGCAATGTCTTGTGGTG |
| comp83411_c1 | Forward | GGATACAGGCTACAATGCAGAGG |
|  | Reverse | GTTAAGGGGCAATACCTTGAGAAC |
| comp76893_c0 | Forward | CTAAGGGATGGTATAACTGGGGAG |
|  | Reverse | CAATCAGAGCCGTCAATGAAGC |
| comp84055_c0 | Forward | GCCATTGACTCCATCCCAG |
|  | Reverse | CGGTCGTGCCATTCAATC |
| comp54935_c0 | Forward | GCAACACCACCTACTACGAC |
|  | Reverse | GGCGGCACTAACATGAAC |
| Actin | Forward | CTCATTGATCGGCATGGAAGC |
|  | Reverse | GGGGAACATAGTTGAACCACCAC |
